# Optimization and Control of Cyber-Physical Vehicle Systems

**DOI:** 10.3390/s150923020

**Published:** 2015-09-11

**Authors:** Justin M. Bradley, Ella M. Atkins

**Affiliations:** 1 Computer Science and Engineering Department, University of Nebraska - Lincoln, 256 Avery Hall, Lincoln, NE 68588, USA; 2 Aerospace Engineering Department, University of Michigan, 1320 Beal Ave, Ann Arbor, MI 48109, USA; E-Mail: ematkins@umich.edu

**Keywords:** cyber-physical systems, control, real-time control, optimization, optimal control, robotics

## Abstract

A cyber-physical system (CPS) is composed of tightly-integrated computation, communication and physical elements. Medical devices, buildings, mobile devices, robots, transportation and energy systems can benefit from CPS co-design and optimization techniques. Cyber-physical vehicle systems (CPVSs) are rapidly advancing due to progress in real-time computing, control and artificial intelligence. Multidisciplinary or multi-objective design optimization maximizes CPS efficiency, capability and safety, while online regulation enables the vehicle to be responsive to disturbances, modeling errors and uncertainties. CPVS optimization occurs at design-time and at run-time. This paper surveys the run-time cooperative optimization or co-optimization of cyber and physical systems, which have historically been considered separately. A run-time CPVS is also cooperatively regulated or co-regulated when cyber and physical resources are utilized in a manner that is responsive to both cyber and physical system requirements. This paper surveys research that considers both cyber and physical resources in co-optimization and co-regulation schemes with applications to mobile robotic and vehicle systems. Time-varying sampling patterns, sensor scheduling, anytime control, feedback scheduling, task and motion planning and resource sharing are examined.

## 1. Introduction

A cyber-physical system (CPS) is “the next generation of system that requires tight integration of computing, communication, and control technologies to achieve stability, performance, reliability, robustness, and efficiency in dealing with physical systems of many application domains” [1]. A cyber-physical vehicle system (CPVS), ranging from automobile to aircraft and marine craft, is composed of tightly-coupled locomotion, computational and communication components. Historically, CPVS development has been driven by advances in closely-related (but not identical) autonomous vehicle research [2,3] and cooperative vehicle control to increase system capacity and improve safety and efficiency [4,5,6,7]. CPS research generally aims to synergistically integrate control, computing, communications and physical systems in novel ways that leverage interdependent behavior.

CPSs have broad applicability and have been the topic of numerous committee workshops and reports [8,9,10,11,12,13] focused on identifying and addressing next-generation opportunities and challenges. Consumer devices, such as smartphones, multimedia players and gaming systems, respond to voice commands, and wearable electronics are ubiquitous. Smart buildings are equipped with advanced sensors, pervasive networking and efficient energy management systems [14]. Advances in medical devices can lower costs and improve patient care [15,16,17,18]. New software-enabled functionality, increased connectivity and physiologically closed-loop systems have the potential to reduce human error that can cost lives [15,19,20]. A new energy service system dubbed the “smart grid” promises to utilize CPS technologies to increase configurability, adaptability, reactiveness and self-manageability [21], but will simultaneously require CPS breakthroughs in security to monitor, manage and thwart threats both to the physical entities comprising the grid, as well as the cyber attacks on its networked components [22]. Most relevant to the work in this paper is the application of CPS research to vehicle systems. In this domain, CPVS research offers an increase in autonomy, reconfigurability, reliability, system capacity, safety, energy efficiency and robustness [23].

Human beings are the quintessential CPSs possessing heavily interdependent cyber (mind) and physical (body) subsystems. Analogous to this mind-body paradigm, advanced CPVSs utilize both cyber and physical resources. However, unlike the symbiotic mind-body awareness humans have, to date, cyber and physical subsystems are unaware or only partially aware of the other. Typically, the cyber system receives performance feedback and calculates trajectories and control inputs for only the physical system. In this way, the cyber system serves the needs of the physical system. This particular role in CPVS has received a great amount of research attention, most prominently in the form of control theory, path planning and real-time system (RTS) theory. It is also likely that the physical system is accomplishing a mission objective that services the goals of the cyber system, for example surveillance, safe transportation, science data collection, *etc*. In this way, the physical system serves the needs of the cyber system. This role, however, has historically been dominated by humans in-the-loop who design high-level plans, set waypoints, control or modify tasks either through an interface or direct software manipulation.

Comprehensive holistic CPVS co-design would account for cyber and physical resources spanning the life-cycle of a system or system of systems [24]. This would include *a priori* co-design of the structure, electromechanical and processing components, baseline software, communication protocols and subsystem composability and compositionality [25]. Holistic CPS run-time system design optimizes and regulates electromechanical and processing element operation in accordance with mission goals and system actuation, processing and energy constraints [26]. While either the *a priori* or run-time CPVS design challenges could serve as a worthy survey topic, this paper focuses on run-time CPVS. Continuing the analogy above, humans are not able to choose their bodies (*i.e.*, the physical “hardware”), and yet, still optimize perception, decision and action for their holistic mind-body system considering both physical (body) and cyber (mind) resources. We consider CPS run-time co-design from the perspective of co-optimizing and co-regulating available physical and cyber resources for a single vehicle after the physical hardware has been manufactured. This means that models and algorithms must capture preexisting characteristics and properties of the physical system, as they are not modifiable.

**Figure 1:**
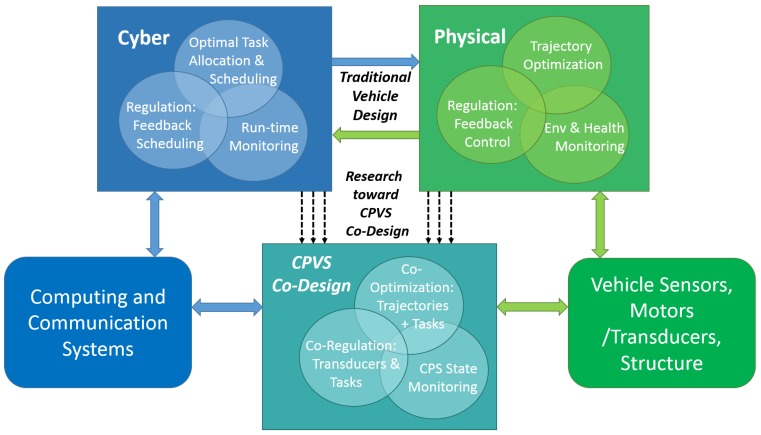
An evolution toward integrated cyber-physical vehicle system (CPVS) run-time co-design.

Emerging CPVS research extends the traditional independently-designed subsystem architectures for modern vehicles toward methods of interdependent and integrated CPVS co-design. In Figure 1, we show the often segregated design techniques contrasted with the tight coupling and integration in a co-designed CPVS.

CPVS research calls for new models, new abstractions, new performance metrics, new design methodologies, new integration methods for large-scale systems, new methods of reasoning about uncertainty and a revolution in how we think about computing [23,27]. While the depth offered by separately modeling and analyzing physical and cyber subsystem behaviors is useful, aberrant system behavior (*i.e.*, when the laws of compositionality or composability do not hold) [25] may be undesirable at best and dangerous at worst. Accounting for as many subsystem interactions as possible can reduce the negative side effects of such behaviors, as well as providing provable holistic system characteristics (e.g., stability) [25]. Integrated analyses and strong co-design through coupling of cyber and physical components can enable more efficient, safe, secure and capable systems as we increase the level of autonomy in CPS devices and vehicles.

This paper contributes a survey of optimization and real-time control or regulation work as it relates to CPVS design. We assimilate work across domains, but focus on aerospace applications and offer a meta-level analysis for how the emerging CPS research community has merged modeling and analysis methods from both cyber and physical perspectives. We offer suggestions about future directions and outstanding challenges to achieve more holistic CPVS co-optimization and co-regulation. Below, we first present a brief history of CPS followed by an overview of modern vehicle design and operational challenges. We then provide a broad review of interdependent co-design techniques for control (regulation) and optimization of CPVS and conclude with thoughts on remaining CPS challenges and research needs.

## 2. History of CPS

The term “cyber,” as a prefix, stems from the field of research known as “cybernetics”, the scientific study of control and communication in the animal and the machine [28]. Cybernetics as a field of research in the modern era began in the 1940s with Norbert Wiener, Warren McCulloch, Ross Ashby, Alan Turing and Grey Walter. Since then, however, semantically, “cyber” is usually associated with information technology, computers and the Internet or to denote control in the computer or electronic context [29]. Perhaps the most fitting definition of the word “cyber” from “cybernetics” stems from Plato’s *The Alcibiades* and is “the study of self-governance” [30]. As a field of research, CPS strives to improve self-governance for machines, infrastructure and devices.

In this paper, we denote as “physical” the tangible actuation, sensing, energy storage, structural and mechanical components of a vehicle system. We use the term “cyber” for the intangible computation and communication functions performed by the CPS similar to the body/mind analogy in animals. This means “cyber” resources refers to available “bandwidth” for computing activities typically in the form of task schedules, utilization of processor cores, *etc*. “Physical” resources are those physical components that host vehicle systems and give rise to locomotion and direct environment interaction (e.g., manipulation) functions.

**Figure 2:**
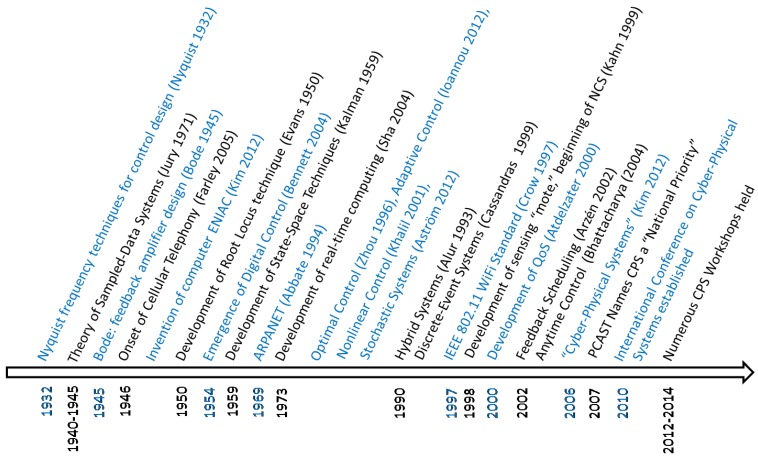
Cyber-physical system (CPS) history.

In the last century, advances in communication, control and computing were primarily used as individual tools in their respective domains. For example, advances in communication were used strictly for communicating between humans without integration into more complex systems. However, as technology has advanced, the integration of the tools and techniques within each domain into more complex systems has provided a new frontier fusing communication, control and computing. An excellent exposition on CPS research and its history can be found in [1]. We highlight select key events on a timeline in Figure 2.

## 3. Cyber-Physical Vehicle Systems

Modern automotive, marine and aerospace applications employ multiple layers of abstraction in software (objects) and hardware (modules) to effect locomotion, internal and external communication and data manipulation, such that mission goals are safely achieved. Autonomy is no longer a question of “if”, but instead a question of “how much”. Each vehicle sector, aerospace, automotive and marine, has wrestled with determining the best levels of autonomy for each mission given current technology and expected operator knowledge and skill.

There are numerous architectures for designing CPVS, often stemming primarily from the robotics community. These architectures are typically a variant of a sense-plan-act paradigm [31], a reactive architecture [32] or guidance, navigation, planning and control loops [33]. The various levels of abstraction in modern autonomy architectures provide corresponding evaluations of performance and system guarantees that have become important indicators of the success of each subsystem. For example, in control theory, stability guarantees are an extremely important indicator of safety for the reactive subsystem. At the planning layer, metrics, such as completeness (guarantees that the algorithm will find a solution if it exists), optimality (guarantees that the algorithm will find the best solution) and complexity (maximum time and memory required by the algorithm to execute), are used to assess performance.

In general, the layers of abstraction in a system architecture can be decomposed into three basic components: the lowest level reactive, control or acting layer, an intermediate execution, guidance or sequencing layer and a high-level planning or deliberation layer [31,32,34]. The reactive or control layer consists of low-level tight control of physical actuators typically employing feedback control techniques. The execution or guidance layer translates higher-level plans into reference trajectories or sequences of actions for the reactive layer. Finally, the planning or deliberation layer consists of a task and motion planning/scheduling algorithm. The planning layer typically requires combinatoric search to optimize a continuous, discrete or hybrid state-space based on goals, constraints and metrics. A co-optimization scheme for planning CPVS tasks and motions generates a desired physical trajectory through space/time along with a real-time computing schedule [35,36,37]. Graph and state-space exploration techniques are employed to generate optimal or desirable paths through a set of discrete states.

### 3.1. Vehicle Control and Optimization Techniques

At its core, CPVSs must achieve their locomotion and task-level mission objectives while meeting safety and efficiency constraints. Planning and control therefore require sensing critical information, selecting actions in the presence of uncertainty and translating high-level plans and objectives into achievable goals for the control and guidance subsystems. Low-level control also requires specifying actuator and configuration commands to all CPVS subsystems. Accomplishing these goals amidst environmental and system disturbances and mitigating the other challenges as described below in Section 3.2 has been the topic of much research in the past century.

Vehicle and environment “robustness” to uncertainties related to external disturbances, sensor noise and modeling errors has been studied across the CPS disciplines. Although the precise metrics for “robustness” in this context vary by research community, a dictionary definition of robustness generally applies across research domains: “capable of performing without failure under a wide range of conditions” [38]. For the physical system safety-critical functionality may require expensive high-quality redundant components with predictable performance. Robust and resilient operation may also require the implementation of physical filtering and fusion schemes using mechanical and electronic components. For cyber systems, it requires digital filtering, data fusion and both retrospective (diagnostic) and predictive (prognostic) analysis of system performance. At each abstraction layer, performance feedback is often used to refine information input to the subsumed layer. Feedback inherently increases robustness to noise and disturbances by using knowledge about the past to decide the future [39].

Digital control is perhaps the most widely-used method for computing feedback control or regulation outputs to a CPVS to provide a robust response to model uncertainties and disturbances. Typically, controllers are designed in either the digital domain directly or in the continuous-time domain and then discretized [40,41]. However, these control systems are usually fixed-gain controllers, which assume worst-case environmental challenges and are, thus, not co-designed with the cyber system in mind.

From the cyber perspective, real-time system (RTS) research focuses on task scheduling, itself an optimization problem, to provide execution guarantees for hard-deadline tasks and best-effort execution for soft-deadline tasks. RTS-centric CPS research has repurposed task execution and scheduling paradigms to accommodate and provide guarantees for classes of tasks appropriate for physically-embodied, highly-dynamic CPS. One example involves characterizing and scheduling tasks with varying execution period [42,43].

Trajectory or physical motion optimization has typically been the domain of continuous-time mathematical analysis, but has also grown to include graph search and probabilistic methods [44]. Motion planning for CPVS generally finds trajectories for the vehicle over a continuous or discretized (waypoint or knot point) state-space that minimizes cost metrics, such as fuel or energy use, elapsed time and safe clearance from obstacles subject to constraints related to system dynamics and consumable resources (e.g., energy). The conventional physical state-space may be augmented or transformed through an appropriate mapping to manage problem complexity [45,46]. Optimal control techniques generate trajectories that minimize costs while meeting constraints using methods, such as co-location and dynamic programming [47].

A number of results from the control and RTS communities have improved our understanding of control and optimization for coupled CPVS. Anytime control [48,49,50], feedback scheduling [50,51,52], networked control system (NCS) [53,54,55], hybrid systems [46,56,57,58], time-varying sampling [59,60,61] and sensor scheduling [62,63,64,65,66] are particularly relevant, covering a spectrum of topics related to an increasingly holistic co-design approach to CPVS. A more thorough discussion of these domains and research can be found in [67].

Because control and optimization of CPVS are the primary subjects of this paper, we describe additional advances to facilitate CPVS co-design and integration in Section 4 and Section 5.

### 3.2. CPVS Co-Design Challenges

Each CPVS domain presents common and unique challenges to CPVS co-design. These challenges can be decomposed into several categories that cut across cyber and physical components of the system. A true co-design will address CPVS challenges across all of the layers, loops and abstractions of the CPVS architecture. Co-design and co-operation challenges cutting across all CPVS domains are discussed below.

#### 3.2.1. Energy Management

Energy management is paramount in any untethered CPVS, as fuel or battery storage resources are almost always scarce. Due to the large power consumption requirements of most CPVS physical propulsion or locomotion mechanisms relative to computational systems, most work to minimize energy use in vehicle systems has focused on minimizing energy used for physical locomotion and actuation. Energy-optimal solutions have therefore focused on optimizing trajectories (physical motions) and feedback control or regulation responses.

Recent technological advances have pushed CPVS toward smaller vehicles with more “intelligence” requirements for payload and vehicle data acquisition, processing and coordination. The reduction in vehicle size has reduced energy requirements for locomotion, while the increase in onboard computing and communication capacity requires more power for components powering computation. The result is that some CPVS now demand as much or more power for computation as for propulsive and other physical actuation systems. This shift, in turn, requires that small data-centric CPVS be co-designed to optimize energy usage over cyber and physical resources. This requires optimizing cyber resources by regulating real-time task schedules, activation of processor cores and communication devices and CPU clock rates.

The automotive sector has been particularly concerned with energy management as hybrid battery/fuel vehicles have emerged [68,69,70,71,72]. In unmanned ground vehicle and field robotics applications, recent work has examined optimal trajectories that account for spatial coverage, energy and time [73,74]. Because energy requirements of propulsive effectors enabling locomotion dwarf other power consumers in commercial automobiles, aircraft, rotorcraft and space launch systems, energy management has typically taken the form of optimal control over the physical vehicle (position, velocity) state [75,76,77]. Small unmanned aircraft system (UAS) and small spacecraft (CubeSat) provide more opportunities to optimize allocation of cyber and physical resources, as will be discussed later.

#### 3.2.2. Fault Detection and Diagnosis

In any CPS, including humans, fault detection, diagnosis and recovery is critical to robust and resilient system operation. These fault management capabilities can be achieved through a combination of hardware for sensing and redundancy and software for data processing and decision-making [78]. Because there is rarely a bijective relationship between signal inputs and decisions, significant computing resources are often dedicated to detecting and then diagnosing problems. On the physical side, fault-tolerant or redundant electronics [79] and sophisticated algorithms based on models of physical systems [80,81] are frequently used.

From the cyber system perspective, software faults, generally exposed by rare events and/or complex function and data interactions [82], are managed by careful design of software [83,84] and explicit fault detection algorithms [85]. Rigorous adherence to good software engineering practices can reduce the likelihood of software faults/failures [82]. Detection and robustness to adversarial attacks on the CPS wherein estimation and control could be compromised are also critical for mission success [86,87].

Interestingly, a software fault detection scheme that is co-designed to detect both cyber and physical faults must simultaneously detect faults in its own algorithm and models thereby complicating co-designed fault detection schemes. However, co-designed fault tolerance methods must be developed to account for the interactions between software-controlled physical and information systems, an important area for future work.

#### 3.2.3. Computational Resource Management

For CPVS with computing resources limited by size, weight, power or some combination, judicious management of memory, disk space, CPU time, communication systems, sensors and actuation is critical given ever-increasing computational requirements to handle *in situ* data acquisition and processing. This is particularly critical or emerging spacecraft, robotic and small UAS applications, where powerful computer components are expensive or impossible due to volume and power limitations.

“Anytime” or “imprecise computing” control techniques offer the benefits of conserving cyber resources at the cost of suboptimal physical system performance [48,49]. In these algorithms, computational resources are traded for suboptimal solutions as needed, and then, solutions are refined as resources become available [50]. Juxtaposed with anytime control, which improves controller decision quality as a function of available deliberation time [48], feedback scheduling dictates the quality of computing of the cyber system by applying feedback techniques to real-time scheduling [88].

#### 3.2.4. Human Interaction

Engineered CPVSs are ultimately designed to support human needs [89]. As a result, even highly-autonomous vehicles will interact with humans at some point, even if only to receive mission goals, then deliver payload or data during or after the mission ends. Automotive [90], aerospace [91] and marine [92] applications have frequent interaction with human operators or passengers, which necessitates consideration of human characteristics, tendencies, limitations, *etc*., in CPVS co-design. Even long-duration spacecraft missions have human scientists as the “end-user” and, therefore, must consider appropriate data formats, communication protocols and operator interactions to maximize mission return.

The human factors research community has studied physical (hardware), cognitive (liveware) and organizational (software) interactions and has made many recommendations [89]. In the aerospace sector, approximately 70%–80% of mishaps are the result of human error, and yet, all too often, investigations are conducted without a “human error framework” to classify human error and recommend appropriate interventions [93]. However, in most vehicle sectors, this issue is being addressed in part thanks to CPS research efforts to devise ways to more closely integrate and monitor human and machine interactions [94,95,96].

#### 3.2.5. Unanticipated Scenarios

The space of possible unanticipated environment and vehicle states is theoretically infinite, while most CPVS are limited to deliberating over continuous and discrete state parameter/value sets that the designer has prescribed *a priori*. As a result, a CPVS may encounter an unanticipated scenario it either cannot sense, recognize or ultimately handle even if recognized. Human beings (and other animals) excel in their adaptability to unanticipated situations through recognizing the novelty of a situation, as well as modifying actions accordingly. Often, the design approach to increase robustness is to anticipate as many scenarios as possible, design with them in mind (e.g., gain-scheduling, state-space partitioning) and then rely on adaptive, uncertainty or learning algorithms to manage scenarios for which a model has been devised, the parameters of which can then be learned [97,98].

More challenging is handling unanticipated situations that can only be accurately represented through the definition of new continuous and/or discrete state features. Emerging machine learning techniques [99] for new state features and model definitions through data clustering, filtering, and understanding show promise for this purpose, but they are not yet sufficiently mature to have been pervasively infused into CPVS. Complementing notions of robustness against unanticipated scenarios are algorithms capable of self-analyzing and self-repairing models to increase autonomy levels in CPVS [100].

## 4. Control of Cyber-Physical Vehicle Systems

Control or regulation of actuators is ubiquitous across CPS generally. For CPVS particularly, in the most general sense, control or regulation makes use of algorithms and feedback to calculate inputs for cyber and physical effectors that provide services, translocate or reorient the vehicle, ensure safety and achieve objectives [101,102]. We refer to holistic, integrated, tightly-coupled CPS modeling and control of both cyber and physical effectors as co-regulation. Here, we focus on a few advances that more holistically consider cyber and physical resources in the co-regulation of effectors.

### 4.1. Anytime Control and Monitoring

Anytime control allows control solutions to be refined or improved as a function of available cyber resources [103]. In some cases, anytime control methods can be shown to be an adaptation of receding horizon control (RHC), wherein a control input is calculated by solving an optimal control problem over a specified time interval [104,105]. Research into the limits of RHC, such as the effects of slow update rates [106], or the bounds on computational time to ensure stability [107], provide an impetus for pursuing anytime control algorithms. We provide an overview of anytime control techniques in [67,108,109] and report here on anytime control and monitoring techniques.

Researchers have investigated algorithms, the solutions of which degrade “gracefully” with reduced cyber resources, and the subsequent usage of these algorithms as building blocks for a real-time system [110]. In this vein, Zilberstein *et al*. provide a comprehensive survey of intelligent systems composed of many “anytime” algorithms [111]. In the context of such a real-time system with many anytime algorithms cyber and physical co-design can be made more robust by providing a supervisory monitor that accounts for uncertainty in the algorithm itself, as well as the cost of monitoring the process [112]. Optimal scheduling of anytime algorithms has also garnered attention [113]. However, much of the difficulty in computational resource management stems from an inability to use such algorithms, as they lack support in many real-time operating systems (RTOSs) and because of their inherent non-determinism [114].

### 4.2. Feedback Scheduling

Feedback, as a principle, offers robustness to off-nominal conditions by using past measurements to compute future inputs to the system and has been applied in many domains. Most related to control of CPVS, and a departure from control of the physical system, is feedback scheduling. Feedback scheduling adjusts cyber resources based on the needs of the cyber system [115]. It accomplishes this by adapting traditional control theory to regulate the task schedule in the RTS. This, in turn, contributes to regulating the CPS as a whole.

In this scheme, sampling periods of various control tasks are adjusted, and subtasks (parts of a task) are scheduled using feedback from execution time measurements and feedforward from workload changes [116]. Cervin *et al*. have developed a sound framework for feedback scheduling of control systems and have provided MATLAB toolboxes for simulation and analysis of real-time control systems [117,118,119,120].

Feedback scheduling algorithms can be computationally intense. A simple (*i.e.*, linear) model that relates the cost of control performance to cyber resources would provide an excellent tool for feedback scheduling, which can then less expensively design task schedules [52].

### 4.3. Time-Varying Sampling and Sensor Scheduling

Uncertainty in sampling rate can be caused by transmission delays in a NCS, jitter and/or missed deadlines in the RTS, *etc*. Research investigating the design of controllers under uncertain delays has resulted in more robust systems. Typically, as in NCS research, these approaches consider a small range of possible sampling rates and stability, and robustness guarantees are given for that range under time-varying control schemes [59]. Successful optimal controllers under these circumstances using a linear matrix inequality (LMI) approach have been designed [60,61].

In many control systems, sensors are read at a higher rate than the control is output to the actuators, guaranteeing up-to-date measurements of the physical system state. Sensor scheduling is a technique used to determine which sensors or sensor modes should be read next to minimize error in the control system [62,63]. This often occurs where many sensors or sensor modes provide readings for similar phenomena. Markov decision process (MDP) formulations typically find an optimal policy for scheduling sensors [64,65,66].

### 4.4. Event-Triggered Control

Event-triggered control, also known as Lebesgue sampling [121], provides an efficient means of achieving acceptable physical system feedback regulation with minimal computational resource overhead. Event-triggered control contrasts with time-triggered or periodic control in that control tasks are triggered and subsequently executed when the system state deviates a certain threshold from a predetermined value [122]. This approach results in more efficient allocation of cyber resources due to “as-needed” or “on-demand” physical system control task execution. While researchers have begun to advance Lebesgue sampling or hybrid approaches to CPS control [43,123,124,125,126], it is a relatively unexplored area of research compared to time-triggered control or Riemann sampling [121]. An excellent introduction to event-triggered and self-triggered control can be found in [122].

### 4.5. Coupled Cyber-Physical Co-Regulation

Finally, our recent co-regulation work is similar to feedback scheduling and time-varying sampling and control. We propose a linear model relating sampling rate to controller performance, which is then added to the linear time-invariant (LTI) model of the physical system [67,108,109,127]. Assuming a physical system modeled as:

$${\dot{x}}_{p}=A_{p}x_{p}+B_{p}u_{p}$$

where ${\dot{x}}_{p}$ is the physical state vector, $A_{p}$ the system matrix, $B_{p}$ the control matrix and $u_{p}$ the physical control input, we seek a system of the form:

$${\dot{x}}_{c}=A_{c}x_{c}+B_{c}u_{c}$$

where components with subscript *c* are the cyber system analogs to the physical model components. This allows us to write the coupled CPS as:

$$\Sigma_{CPS}:\left\{\left[\begin{array}{c} {\dot{x}}_{p}\\ {\dot{x}}_{c} \end{array}\right]=\left[\begin{array}{cc} A_{p} & \\ & A_{c} \end{array}\right]\left[\begin{array}{c} x_{p}\\ x_{c} \end{array}\right]+\left[\begin{array}{cc} B_{p} & \\ & B_{c} \end{array}\right]\left[\begin{array}{c} u_{p}\\ u_{c} \end{array}\right]\right.$$

**Figure 3:**
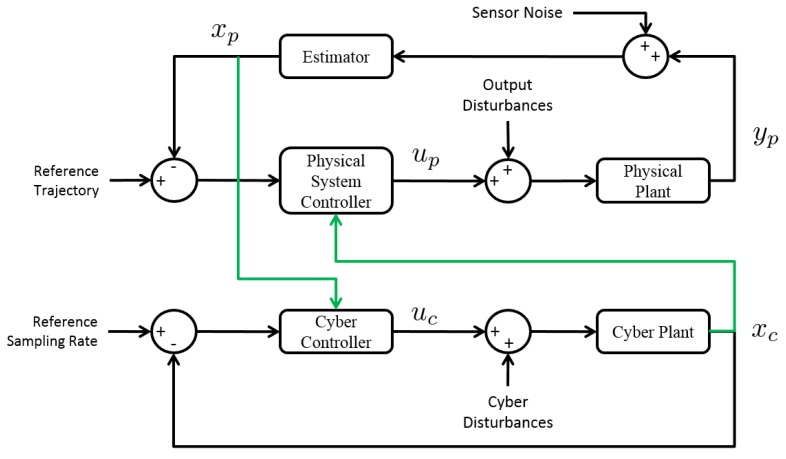
CPVS co-regulation scheme.

In Figure 3, we depict this co-regulation scheme as a traditional control systems block diagram.

The physical and cyber systems are modeled independently, but coupled through feedback control. State estimates of the physical system are leveraged in the cyber controller, and cyber states are used to calculate the discrete-time-varying physical system controller.

In [67,127], using feedback control techniques, we design two discrete-time varying controllers for the physical system. The first utilizes gain-scheduling, where controller gains are “scheduled” (Note that this is not the same as RTS task scheduling, but rather controller gains are selected at each time step for the system linearized over various operating points.) over sampling rates of the control task. The second controller uses forward-propagation Riccati-based control to propagate the control gain from one discrete time step to the next. We then design controllers for the cyber system, which adjust the control task execution rate (*i.e.*, sampling rate) according to the error in tracking the reference trajectory. Finally, we introduced CPS metrics to measure important physical and cyber performance. These metrics capture a portion of RTS utilization, the error of the system trajectory with respect to its reference value and the control effort of the physical actuators. We used these metrics in a CubeSat CPVS co-regulation application to provide a domain-inspired characterization of potential savings in control effort and to make cyber utilization realizable [127].

This co-regulation scheme has illustrated CPS tradeoffs possible over a series of domains including a spring-mass-damper system [108] and inverted pendulum [109], as well as the CubeSat domain [127]. In all cases, co-regulation consistently demonstrated good physical system tracking performance, while significantly reducing computational load. This abstraction approach to CPS co-regulation allows an engineer to leverage the wealth of traditional state-space control design techniques and to treat the scheduling of tasks as a control problem wherein interactions between cyber and physical states are represented in a common framework. It also provides the benefits of time-triggered control, such as ease of RTS scheduling and hard timing guarantees, while also offering the benefits of “on-demand” event-triggered control to reduce cyber resource utilization.

This work is distinct from feedback scheduling in that we seek to couple the resulting cyber system “dynamics” model to the physics-based model, so that both can be co-regulated in each execution of the control task. This also allows us to apply feedback control techniques and analysis to the coupled CPS. This work is similar to optimal sampling pattern techniques (discussed in Section 4.3 in that it allows for variable sampling instants. However, whereas that work focuses on optimality over a planned trajectory, our technique focuses on increasing robustness to system disturbances and deviations from planned trajectories through proportional feedback control, which determines the sampling rate. Additionally, our feedback co-regulation scheme could be used to supplement optimal sampling pattern techniques by accepting the optimal sampling pattern as the reference trajectory and using feedback co-regulation to offer minor adjustments based on off-nominal conditions.

## 5. Trajectory and Task Optimization and Planning for Cyber-Physical Vehicle Systems

Optimization is fundamental to most engineering disciplines. Quite generally, an optimization process finds values over a design vector or state-space that maximize utility (or minimize cost) subject to specified constraints and system dynamics [44,128,129]. Engineering optimization is pervasive to system design, analysis and operation. Optimization has evolved from a time where human engineers would specify small-scale subsystem optimization problems to multidisciplinary optimization efforts where a vehicle or other complex system is co-designed with respect to a potentially large suite of heterogeneous design parameters [130,131]. As such, optimization is a natural tool for CPS. In this paper, we refer to the holistic application of optimization to CPS as co-optimization with a focus on CPVS. Below, physical trajectory and real-time computing optimization efforts are summarized, followed by a discussion of emerging work in co-optimization.

### 5.1. Physical Trajectory Optimization

A “physical” optimization problem is typically described by a state-space $\vec{x}\left(t\right)$ of CPS platform positions and velocities, as well as any moving subsystems (e.g., a gimballed payload). The cooperative team state can be described by a family of state-space vectors $\vec{x_{j}}\left(t\right)$ for each vehicle *j* in the team. Network connectivity for cooperative planning and control [132,133] can be described by a graph Gx with edges $Ex_{i,j}$ between vehicle pairs (i,j) that directly communicate. Vehicle dynamics are given by the physics-based equations of motion, while network connectivity is usually a function of physical separation distance. Vehicles can carry sensors and/or deploy immobile sensors; vehicles and immobile sensor nodes both host processing and communication resources. The CPS community has investigated how to best optimize CPVS team physical trajectories (CPVS positions, motions) to best support networking [134] and to optimize information acquired by a sensor CPVS team [135]. For example, Song *et al.* [135] propose use of the Fisher information matrix to optimize observations over a wireless sensor network (static or mobile). This manuscript focuses on the CPVS challenges faced in a single CPVS rather than a networked team, with full recognition that CPVS networks present a suite of distributed physical, processing and communication challenges also important to recognize and address. For rigid body vehicles, such as cars, planes or mobile robots, optimization generates a potentially long-term path or trajectory designed to accomplish the mission while meeting physical actuation, collision and sensing constraints while minimizing costs, such as consumed energy and traversal time. Geometric solutions minimize traversal length as in the Dubins solution [136] for turning-constrained vehicles applied to many domains [137,138] with several extensions [139,140]. Geometric solutions are computationally-efficient to find, but difficult to generalize to new objectives. Computational optimal control [44,141] has also been frequently applied to physical vehicle trajectory optimization based on necessary conditions for optimality [142,143]. While optimal control can be computationally expensive and solution convergence difficult to guarantee, optimal control methods provide a fully general means of identifying optimal trajectories over a design (state) vector with generalized cost terms and constraints. Time and energy costs are most frequently defined for physical vehicle trajectory optimization [144,145,146], and methods, such as nonlinear programming [147] and quadratic programming [148], have been applied.

Geometric and optimal control methods can solve boundary value problems (BVPs) where a vehicle travels to a single destination. Optimal physical trajectories can also be computed through multiple goal or destination points, the traveling salesman problem [149,150]. Solution methods, such as dynamic programming [151,152], have been developed with many variants. The Bellman equation [151] considers immediate and expected future reward to optimize actions in a discrete state-space. The introduction of uncertainty gives rise to Markov chain [153] discrete state dynamics for which stochastic dynamic programming (SDP)/MDP methods can be applied to generate optimal policies [154]. Randomized algorithms, such as genetic algorithms, simulated annealing and particle swarm optimization [129] can explore large search spaces.

### 5.2. Computing (Cyber) System Optimization

The real-time systems community has also capitalized on optimization techniques to ensure that computing and networking systems are efficiently utilized. A real-time or “cyber” task allocation and scheduling problem is defined over tasks i∈τ with processing and memory load requirements $p_{i}$ and $m_{i}$, respectively. Communication over a distributed processing system can be described by graph Gc with edges $Ec_{i,j}$ between processor pairs i,j that directly communicate. Multi-core systems can assume full connectivity and shared memory, while distributed computing systems must optimize task allocations over required data transfer and connectivity constraints, as well as processor load balancing.

Task allocation and scheduling methods optimally distribute hard and soft real-time computational tasks over single-core and multi-core processing architectures [115,155]. Metrics include task priority, expected or worst-case execution time and memory, arrival time and hard or soft real-time deadline. Schedulers, such as rate-monotonic [156] and earliest deadline first [157], laid a foundation for the mathematics of scheduling. Optimal solvers also exist for multi-processor [158] and GPU [159] architectures. Networked CPSs have been studied from the perspective of optimally positioning and moving wireless sensor network elements as discussed above. From the cyber perspective, the CPS workload to be optimally managed across the team is defined as “the amount of measured and/or processed data per unit of time that is communicated between various CPS nodes [CPVS] and which affects not only various local parameters (e.g., buffer utilization), but also macroscopic metrics (e.g., CPS throughput)” [160]. A cyber-oriented CPVS optimization approach would therefore minimize CPS workload while also ensuring that computational resources onboard each CPVS are not over-utilized given necessary vehicle-specific real-time tasks plus network-based workload.

### 5.3. Co-Optimization

A rich set of CPS optimization methods have arisen from the “physical trajectory optimization” and “real-time computing” communities. The mathematics of CPS optimization shares a common heritage motivating our focus on CPS optimization problem formulation rather than devising new solution methods. Physical trajectories are now beginning to consider cyber costs related to limits in data acquisition and onboard processing for applications, including sensor networks [161] and cooperative motion planning [25]. Real-time embedded computing has been extended to CPS [162] by considering the physical environment in cost and constraint formulations, using processor voltage scaling and core shut-down to manage computing power [163] and thermal loads [164] with constraints related to component reliability [165].

In the context of the UAS, missions have been optimized by leveraging optimal control to design physically-efficient trajectories that satisfy actuator and structural constraints [166]. Trajectory costs include mission time [167] and total energy consumption [168]. UAS cyber systems are typically optimized separately to ensure that real-time computational and information-sharing task deadlines are met. The primary real-time computing cost term is processing and communication system energy use [169,170,171].

In our previous work [35], we optimized a UAS inspection mission over an integrated CPS cost function, including mission time, physical actuator and computing system energy consumption, payload information (gaps) and processor utilization. More recently, we added physical (battery, motor) and computing system thermal costs and constraints [172].

This has laid a foundation for recent and future CPS optimization research. For example, processor power and thermal regulation [164] consider physical environment impact on cores, but do not optimize physical mobility/actuation (e.g., by demanding less torque, thereby lowering heat production in the (self-driving) car’s engine compartment). Similarly, in our own work [35,172], a UAS trajectory is itself optimized over cyber-physical terms, including the energy used and heat generated by propulsion and computing systems as a function of physical state and processor utilization trajectories. To offer the benefits of dynamic sampling alongside the critical guarantees of time-triggered sampling, very recently, optimal sampling and control techniques have arisen. Bini *et al.* proposed an optimal control formulation that simultaneously optimizes control inputs and sampling pattern trajectory. They also proposed a computationally-feasible quantization-based method to approximate the optimal control solution and proved optimality for first order systems [173]. Kowalska *et al.* recently proposed varying time control (VTC), a similar optimal control problem over control inputs and sampling instants. In this formulation the optimal control problem is solved for a receding horizon with a computationally tractable algorithm [174], but loss of optimality guarantee [173].

### 5.4. Co-Optimization Example

Cyber and physical optimizers often minimize time and energy (power) use while maximizing the accomplishment of mission objectives. Mobile systems, such as UAS, carry limited energy resources, resulting in hard energy constraints, while vehicle stability, environmental hazards, *etc*., impose real-time transit and computing constraints. In our previous work [35,172], we developed a suite of physical, cyber and combined cost function to optimize UAS pipeline inspection. A gimbal-mounted camera onboard a UAS provided imagery, which was processed by a real-time task. The objective was to minimize cost as a function of aircraft airspeed and execution rate of the image processing task. We briefly describe this approach to offer an example of how a co-optimization scheme may be designed.

Let $J_{p}$ and $J_{c}$ represent physical and cyber cost terms, respectively. The CPS optimizer cost function must then minimize total integrated cost:

(1) $$J_{CPS}=J_{p}+J_{c}$$

Physical cost, $J_{p}$, includes time, *T*, and energy, $E_{p}$, cost terms:

(2) $$J_{p}=\beta_{p2}T+\beta_{p1}E_{p}$$

where $\beta_{p1}$ and $\beta_{p2}$ are weighting terms. Time, *T*, and total energy, $E_{p}$, consumption by physical systems (servos, motors) over the mission to final time $t_{f}$ are given by:

(3) $$T=\int_{0}^{t_{f}}dt,\;E=\int_{0}^{t_{f}}P_{p}\left(v\left(t\right)\right)dt$$

A single real-time task, τ, executes at variable rate $r_{\tau }\left(k\right)$ to acquire and process payload images. Cyber cost, $J_{c}$, consists of a term proportional to real-time utilization, $U_{\tau }$, and an entropy-inspired term, *H*, which gives a measure of information gathered about the pipeline:

(4) $$J_{c}=\beta_{c1}U_{\tau }+\beta_{c2}H$$

where $\beta_{c1}$ and $\beta_{c2}$ are weighting terms. We have assumed that power use is proportional to total cyber utilization cost

(5) $$U_{\tau }=\sum_{k=1}^{N}\frac{r_{\tau }\left(k\right)}{r_{\tau ,\max}}$$

where *N* is the total number of task execution cycles, *k*, in the mission. The information term, *H*, couples UAS airspeed, v(t), and task execution rate, $r_{\tau }\left(k\right)$, as the UAS. A measure of scene overlap in the image, Ω, reduces entropy by providing pipeline data from multiple perspectives $\vec{x}\left(t\right)$:

(6) $$H=\frac{1}{N-1}\sum_{k=2}^{N}e^{-\alpha \Omega (v(t),k)}$$

See [35,172] for additional details of $J_{CPS}$ and Pareto front analyses.

## 6. Discussion

Above, we have described run-time CPVS co-optimization and co-regulation from the dual perspectives of the physical control and real-time computing communities. Because CPS represents an emerging interdisciplinary community, CPS constituents necessarily offer methods that most naturally apply to problems rooted in the researcher’s particular background and experiences. While reading the above content, a CPS researcher with a control theory background may have thought primarily about the stability and convergence properties of the co-regulation scheme summarized above and envisioned an optimal sequence of equilibrium states and accelerated maneuvers for the co-optimized CPVS trajectory. A CPS researcher with real-time computing background may have thought critically about the fact that we have, to-date, only co-optimized over a single payload data processing task [175] and co-regulated over a single “cyber task” associated with feedback control [127], whereas a typical embedded real-time computing system would optimize and regulate a far larger suite of soft and hard real-time computing and communication tasks.

In future co-optimization research, it will be important to extend the single-vehicle CPS to consider the suite of additional processing and communication tasks required for a complex vehicle system, such as an automobile or aircraft, to capably manage all of its embedded resources, as well as its motions and (low-bandwidth) contact with “the cloud” and other networked vehicles. Co-optimization can also be extended to networked CPVS teams to ensure that information collection, energy use, processing and communication resources are all managed optimally while respecting individual CPVS energy, time and physical environment (e.g., collision) constraints. Similarly, future co-regulation research for a single vehicle must consider the management of auxiliary physical controls, e.g., a payload gimbal, as well as primary rigid-body motion controls; the “cyber” state considered during co-regulation must, in turn, regulate planning, guidance, navigation and payload (data) real-time processing and communication processor and communication utilization along with feedback control task rate regulation. This extension might be mirrored in the context of local regulation for cooperative control, e.g., consensus [133], coupling the local coordination of motion with real-time data acquisition and management across a CPVS team. The CPS/CPVS community is approaching a critical point where dual consideration of physical and cyber metrics and constraints will become commonplace, both for individual CPVS and teams.

As CPS education programs emerge, so will the level of integration in research efforts. Meanwhile, this paper and others like it will continue to encourage the community to think deeply about challenges and potential solutions that cut across the various fields that have contributed to CPS and CPVS. As summarized above, the control theory community has embraced state machine and graph-based models, offering improved representation and analysis techniques for motion planning, feedback stabilization and regulation and cooperative control systems that now consider switched dynamics and discrete task-level or information-centric mission goals. These modeling and analysis methods are valuable to CPS researchers from all backgrounds. Similarly, computer scientists have extended their analyses to better consider aspects of physically-mobile systems with respect to the impact on embedded computation and communication elements. This has resulted in improved modeling, analysis and optimization/scheduling techniques that take into account physical vehicle motions and system dynamics, resource constraints and environment characteristics. Collectively, the embedded control and real-time computing communities offer a suite of complementary methods, but work is still ongoing to deeply understand how CPS can further mature to achieve the ideal “co-design” concept originally illustrated in Figure 1.

A CPVS is distinct from an autonomous system in that the CPVS may or may not be directly controlled by the human operator/driver. Any safety-critical CPVS must be robust and resilient, yet an expendable CPVS, such as a small UAS or CubeSat, may present no threat to people or property of value, enabling tradeoffs between cost and complexity with resilience. A CPVS may be special-purpose, for example a small UAS that can only carry a camera payload or a car with sensors that only communicate in a WiFi-enabled region. On the other hand, the CPVS may require substantial adaptability, motivating the need for capable and reconfigurable processing, sensing, communication and actuation systems. The notions of co-optimization and co-regulation apply to any of the CPVS concepts referenced, although the metrics, constraints and robustness requirements will be tailored to the needs of each application.

Thus far, the CPS and CPVS communities have focused on the real-time operation of a system with a given design operating in a well-modeled environment. This paper has briefly discussed the extension of CPVS to support fault management, parameter adaptation when models are imprecise or incorrect and, ultimately, online data processing to provide support for managing unanticipated events. The traditional engineering community is contributing a variety of sensor data filtering and processing methods, whereas the computer science community is contributing a variety of big data learning methods to extract parameters and models from large datasets. The next generation of CPVS will be even more capable, as these two groups build stronger ties, ultimately merging the physical (sensor) and information (database) sources into new CPVS modeling and analysis tools.

In previous work, we developed and made use of several new CPVS metrics to assess performance [35,36,67,127]. These metrics serve to illustrate how holistic CPVS performance might be quantified in relation to a few possible measures. Additional innovative CPVS metrics are needed to fully capture performance and safety. Learning or adaptive algorithms are desirable for their ability to deal with unanticipated situations, uncertainties and non-determinism. This, in part, excludes adaptive systems from infusion in safety-critical vehicle systems, as they are difficult to certify through traditional software engineering validation and verification processes. The aerospace community, in particular, has a strong record in safety, and pressure is being increasingly applied to make automotive vehicles safer for drivers, passengers and property. However, these systems must rely on human operators and drivers, who themselves are best modeled as uncertain systems, until adaptable methods can be prominently used in increasingly autonomous CPVS. This challenge must be resolved by researchers, policy-makers and through public acceptance and trust with the end goal of establishing acceptable levels of safety for CPVS reflected in new metrics for CPVS performance and safety analyses.

## 7. Conclusions

This paper has surveyed the literature describing methods to model and analyze CPVS with a focus on optimization and feedback-driven regulation of real-time embedded CPVS. While the literature considers a variety of CPVS applications, examples in this paper focused on aerospace applications to motivate how co-designed CPVS can effectively integrate methods devised across the existing communities of practice, in particular to incorporate critical research products from real-time computing and physical control system fields.

Existing efforts to model and analyze systems using graph-theoretic formulations to supplement continuous-state models now support modeling and analysis of CPVS through augmented state machine models. Extensions to handle network, multi-core and multi-vehicle systems further extend applicability. These methods were reviewed and followed by a deeper study of co-optimization and co-regulation capabilities for CPVS. The paper culminates with a discussion of ongoing CPVS challenges.

The CPVS/CPS community faces substantial challenges in research, education and, ultimately, acceptance. How can future engineers and computer scientists be trained to think most clearly about CPS and CPVS? How can CPS and CPVS be even more holistically co-designed and analyzed despite the increasing complexity associated with integrating design analyses across cyber and physical system models? How can such systems be deployed, operated and ultimately trusted to be effective and safe for human users? The answers to these questions will only become clear as CPS technologies and, indeed, the holistic CPS community-of-practice further mature.
